# A Survey on the Computational Approaches to Identify Drug Targets in the Postgenomic Era

**DOI:** 10.1155/2015/239654

**Published:** 2015-04-28

**Authors:** Yan-Fen Dai, Xing-Ming Zhao

**Affiliations:** ^1^Institute of Systems Biology, Shanghai University, Shanghai 200444, China; ^2^Department of Mathematics, Shanghai University, Shanghai 200444, China; ^3^Department of Computer Science, School of Electronics and Information Engineering, Tongji University, Shanghai 201804, China; ^4^Key Laboratory of Systems Biology, Shanghai Institutes for Biological Sciences, Chinese Academy of Sciences, Shanghai 200031, China

## Abstract

Identifying drug targets plays essential roles in designing new drugs and combating diseases. Unfortunately, our current knowledge about drug targets is far from comprehensive. Screening drug targets in the lab is an expensive and time-consuming procedure. In the past decade, the accumulation of various types of omics data makes it possible to develop computational approaches to predict drug targets. In this paper, we make a survey on the recent progress being made on computational methodologies that have been developed to predict drug targets based on different kinds of omics data and drug property data.

## 1. Introduction

In the past decades, the time and cost of developing new drugs have soared significantly. In general, it takes about 15 years and up to 800 million dollars to convert a promising new compound into a drug in the market [1]. In the procedure of drug discovery, the identification of drug targets is the first and one of the most important steps. With the therapeutic targets, the optimal compounds with expected effects can be designed and new indications of old drugs may be discovered. For example, mitoxantrone was originally designed as a type II topoisomerase inhibitor. Recently, Wan et al. [2] found that mitoxantrone can inhibit the PIM1-mediated phosphorylation in cancer cells by binding to PIM1 kinase. Another example is ellipticine that was designed to target Top2 protein, but recent* in vitro* experiments indicate that ellipticine is able to decrease the proliferation rate in cancers by selectively targeting Pol-1 [3]. The targets of drugs also provide insights into the mechanism of actions (MOAs) of these drugs. Therefore, large efforts have been made to screen drug targets in lab. Accordingly, the information about drug targets has been deposited in many public databases (see Table 1), for example, STITCH [4] and DrugBank [5]. These valuable resources make it much easier to design new drugs. However, the knowledge about drug targets is far from comprehensive, which hampers the discovery of new drugs. Considering the cost and time spent in searching for drug targets, it is not feasible to screen all possible molecules targeted by drugs in lab.

Under these circumstances, some computational approaches have been proposed to identify or predict drug targets* in silico*. In particular, the accumulation of various types of omics data, such as gene expression and protein structure, makes it possible to develop more efficient computational methodologies to predict drug targets. For example, with the assumption that the drugs with the same MOAs will induce similar gene expressions, Iorio et al. [6] proposed a new approach to identify drugs that may target the same proteins. Assuming that drugs with similar MOA bind to similar pockets on the protein surfaces, some computational approaches have been developed to predict drug-protein interactions by investigating the similarity between binding profiles of candidate ligands and known drugs [7, 8]. Supposing that proteins with similar functions may be bound by same drugs while drugs with similar chemical structures possibly target same proteins, Yamanishi et al. [9, 10] proposed a novel model to predict drug-protein interactions by integrating chemical structure and genomic sequence information, and they later further took into account the pharmacological information to improve prediction accuracy.

In this review, we present the recent progresses on computational methodologies that have been developed to identify drug targets. In particular, we focus on those methodologies based on gene expression data, molecular networks, and pharmacological information due to the rich resources of these types of data. As a well studied topic, those computational approaches that have been developed to predict drug targets based on protein structures are referred to in a recent review paper by Tan et al. [8]. Furthermore, we introduce popular public resources about drug target information, which can significantly facilitate the discovery of new drugs. Note that this survey aims to summarize the recent progress on computational approaches for prediction of drug targets; however, it is by no means comprehensive due to the rapid evolvement of the field.

## 2. Predicting Drug Targets Based on Gene Expression Profiles

**Figure 1:**
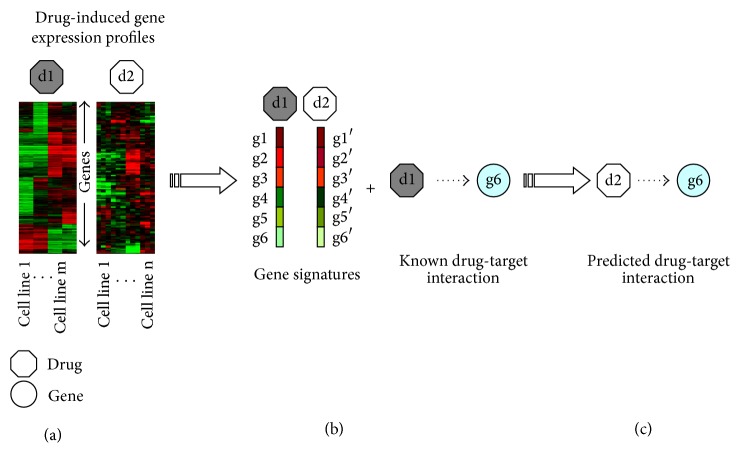
A schematic view of identifying drug-target interactions based on drug-induced gene expression profiles. (a) The drug-induced gene expression profiles across cell lines. (b) Define a gene signature for each compound and calculate the MOA similarity between each pair of drugs. (c) Predict targets for novel drugs with the assumption that drugs with similar MOAs are likely to target same proteins.

A large part of known drugs target certain proteins to exert their functions after they are administered. Therefore, the gene expression profiles induced by drugs can provide insights into the mechanisms of action of these drugs to some extent, where the transcriptome data is able to monitor the expression dynamics of tens of thousands of genes simultaneously. Recently, the publicly accessible gene expression profiles, for example, Connectivity Map (CMap) (http://www.broadinstitute.org/ccle/home), NCI-60 cell lines (http://dtp.nci.nih.gov/), LINCS (http://lincs.hms.harvard.edu/db/), and CCLE (http://www.broadinstitute.org/ccle/home), make it possible to predict drug targets based on the transcriptome data. As shown in Figure 1, some computational approaches have been presented to define expression signatures that are able to characterize the MOAs of corresponding drugs, and these signatures can in turn be utilized to predict targets of novel compounds, where it is assumed that the drugs binding to the same proteins will induce similar gene expression profiles.

In their pioneering work, Lamb et al. [11] established the CMap (Connectivity Map) database that is composed of the genome-wide gene expression profiles induced by more than one thousand compounds across four cell lines. Furthermore, they defined gene signatures from these expression data to characterize the MOAs of those compounds and in turn utilized these signatures to connect small molecules with genes and diseases. The new indications of some drugs were discovered based on the alignment of drug signatures with the assumption that drugs with similar signatures may have similar therapeutic effects [12]. Based on the gene expression profiles from CMap, Iorio et al. [6, 13] constructed a drug-drug network (DDN), where drugs with similar signatures were connected. Furthermore, they extracted network communities from the DDN, and drugs with similar MOAs were found to be enriched in each community. Accordingly, the drugs in the same community are more likely to target the same proteins or pathways. They provided a computational tool, called MANTRA (http://mantra.tigem.it/), to facilitate the analysis of drug-induced gene expression profiles. Iskar et al. [14] presented a new strategy to normalize the gene expression profiles from CMap, which significantly removed the batch effect inherited in the datasets. With the signature defined similar to GSEA [15] for each drug, they successfully identified drugs with similar mechanisms and found new targets for some drugs. Analysis of characterized modules constructed with drug-induced coregulated genes reveals that zaprinast, a drug that had been previously reported to be clinically unsuccessful, is refereed interacting with new target PARA*γ* and has been experimentally validated successfully [16].

The NCI-60 cell line dataset [17] generated by the Development Therapeutics Program of the National Cancer Institute (NCI) is another commonly used valuable resource that contains expression profiles of genes and miRNAs induced by ~400,000 compounds across 60 cell lines. With the assumption that compounds with similar activity profiles may target similar proteins, new possible drug-protein interactions can be predicted by clustering analysis of compound bioactivity profiles across cell lines. To facilitate discovery of anticancer drugs based on the NCI-60 dataset, Reinhold et al. [18] developed a web-based tool called CellMiner along with the expression profiles of 22,217 genes and 360 microRNAs across 60 cell lines perturbed by 18,549 compounds. They identified Tdp1 as the new target of indenoisoquinoline that was originally thought to target Top1 only [19]. Yan et al. [20] identified thioredoxin reductase as a potential target of indolequinone by screening drugs in pancreatic cancer cell line and compared the compounds' bioactivity profiles against those from the NCI-60 cell line panel. Cheng et al. [21] presented a computational approach, namely, BASS, to calculate drug similarities based on their bioactivity profiles, which can in turn be utilized to predict new target(s) for known drugs or targets for novel compounds.

Beyond the above compound-centered large datasets, the accumulation of huge amount of gene expression profiles deposited in the Gene Expression Omnibus (GEO) also significantly facilitates the identification of drug targets. For example, utilizing the transcriptome profiles treated with letrozolein, the ER^+^ breast tumors, Penrod and Moore [22] proposed an influence network approach that can not only identify promising targets but also suggest potential target combinations. The publicly available huge amount of transcriptome data is making it an attractive field to predict drug targets and reposition known drugs based on the gene expression profiles. In addition, the genome-wide gene expression profiles provide new insights into the drug MOAs from a systematic perspective.

## 3. Identifying Drug-Target Interactions from Molecular Networks

Despite the usefulness of the transcriptome data, most drugs exert their functions by affecting the activity of proteins, whereas it is known that there is a gap between the transcriptome and proteome [23]. The biological systems consist of various molecular interactions, for example, protein-protein interactions, and these interactions can be represented as distinct molecular networks depending on the interaction nature. The molecular networks can provide insights into the context in which the drug target works and can therefore help understand the drug mechanisms of action.

**Figure 2:**
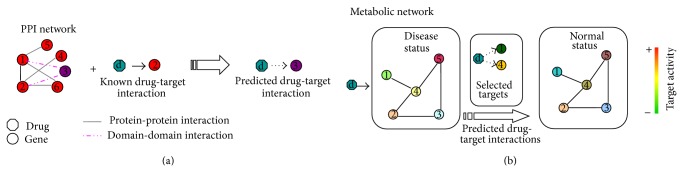
A schematic view of identifying drug-target interaction from molecular networks. (a) Identify drug targets from PPIN supposing that proteins in close proximity of the PPIN are more likely targeted by the same drug(s). (b) Predict drug targets based on metabolic networks assuming that the targets are able to interrupt the pathological procedure so that the disease status can be reversed to normal status.

Among various types of molecular networks, the protein-protein interaction network (PPIN) is well studied. Since the PPIN provides the context in which the target protein works, the PPIN is also utilized to predict drug targets with the assumption that the proteins targeted by drugs of similar MOAs tend to be functionally associated and be close in the PPIN [24, 25]. As shown in Figure 2(a), if a protein is close to the one targeted by a drug, this protein is more likely to be targeted by the drug or a drug with similar therapeutic effects. Based on this idea, Zhao and Li [26] proposed a novel method named drugCIPHER to predict drug-target interactions by integrating drug therapy information, chemical structure information, and PPIN. Later, drugCIPHER has been successfully applied to predict targets of traditional Chinese medicine (TCM). For example, AKT and SRC were identified as targets of vitexicarpin [27], CCR2 was identified as the target of three compounds betulin, fucosterol, and amyrin [28], and IL1R1 was the target of matrine, a bioactive compound of the herbal formula Qing-Luo-Yin [29]. Considering that some nodes play more important roles than others in a complex network, some computational approaches have been proposed by taking into account some network attributes, for example, degree and centrality, to characterize the drug targets. The degree of a protein in the PPIN is the number of interactions in which this protein is involved, while centrality indexes quantify the relative importance of a protein. For instance, Yao and Rzhetsky [25] utilized the protein betweenness centrality in a PPIN to predict drug-target interactions (DTIs) with the assumption that good targets should be of low “betweenness centrality” since the interruption of those highly connected nodes in the PPIN may cause broad and often unintended consequences. Hwang et al. [30] investigated DTIs from the perspective of bridging centrality. Using degree and centrality as features, Zhu et al. [31] trained a SVM classifier to rank potential drug targets and achieved promising results. Among their top 200 predictions, 94 proteins were validated as drug targets in DrugBank [5] database while some novel predictions can find supporting evidences in literature and other public databases.

Considering that the structure and function of a protein are generally determined by its component domains, we proposed a novel computational approach to predict drug targets supposing that drug-protein interactions are dominated by drug-domain interactions even if the drug-domain interactions are not necessarily physical binding interactions [32]. In our approach, the drug-domain interactions were first inferred from known drug-protein interactions as below:

(1) $$\begin{array}{c} P\left(m_{i}\_d_{{\text{ATC}}_{j}}\right)=\frac{N\left(p\;|\;m_{i}\right)}{N\left(p^{\prime }\;|\;m_{i}\right)}, \end{array}$$

where ATC code is the abbreviation of “Anatomical Therapeutic Chemical,” a classification system used for the classification of drugs, ATC_(*j*)_ means ATC code *j*,  *P*(*m*_*i*__*d*_ATC_*j*__) is the probability that domain *m*_*i*_ interacts with drugs annotated with ATC_*j*_,  *N*(*p*∣*m*_(*i*)_) denotes the number of proteins that are bound by drugs belonging to ATC_*j*_ and contain domain *m*_*i*_ as well, and *N*(*p*′∣*m*_*i*_) is the number of all human proteins that contain domain *m*_*i*_. After obtaining the probability of drug-domain interactions, we can determine whether a pair of drugs and domain interact by setting a threshold, where those drug-domain pairs with probabilities above the threshold were treated as drug-domain pair interactions. Accordingly, we can predict drug-protein interactions based on the drug-domain interactions as follows:

(2) $$\begin{array}{c} P\left(p_{i}\_d_{{\text{ATC}}_{j}}\right)=1-\prod \left(1-P\left(m_{k}\_d_{{\text{ATC}}_{j}}\right)\right), \end{array}$$

where *P*(*p*_*i*__*d*_ATC_*j*__) is the probability of protein *p*_*i*_ interacting with drugs belonging to ATC_(*j*)_,  *P*(*m*_*k*__*d*_ATC_*j*__) is the probability that domain *m*_*k*_ interacts with drugs from ATC_(*j*)_, and *p*_*i*_ is a protein that contains domain *m*_*k*_. The results on benchmark dataset show that our proposed approach can improve prediction accuracy compared with other popular methods. Later, with the drug-domain interaction network, Moya-García and Ranea [33] found that drugs are organized around a privileged set of druggable domains, which can help explain drug polypharmacology.

Except for PPIN, the metabolic networks are also widely used to predict drug targets. In the metabolic network based approach, it is assumed that the disruption of pathogenic pathways or inhibition of certain molecules can help reverse the disease state to normal state. With flux balance analysis (FBA) of metabolic networks, Li et al. [34, 35] developed a new approach to identify potential therapeutic drug targets by comparing the fluxes of reactions and metabolites in pathologic and medication states based on linear programming. By simulating the flux distribution in the metabolic network, Folger et al. [36] successfully identified some targets of anticancer drugs. With a detailed disease network, Yang et al. [37] proposed a computational framework to identify optimal multiple target intervention (MTOI) solution by simulating the dynamics of the system with mass action modeling along with simulated annealing. The optimal target combinations detected by this promising method not only overcome the compensatory mechanisms in diseases but also avoid unwanted side effects caused by possible off-targets. By integrating the gene expression profiles across cell lines and human metabolic networks, Li et al. [38] identified new enzyme targets with kernel *k*-nearest neighbor (kNN) classifiers by comparing the reaction flux of novel compound-reaction against that of known drug-reaction. Furthermore, utilizing the genome-scale metabolic models (GSMMs), Yizhak et al. [39] proposed a metabolic transformation algorithm (MTA) to search for targets that could restore the metabolism within the cell from the source (disease) state to the target (healthy) state.

Since the molecular networks are able to provide the circuit context in which the drug target protein works, they provide a straightforward way to understand how the drugs affect or regulate the biological systems. Unfortunately, our current knowledge about the molecular interactomes at different levels is far from complete. Even though large-scale interactomes have been detected or predicted, they are just static snapshots of the biological systems, whereas the real biological systems are spatially and temporally dynamic. Furthermore, little is known about the detailed interaction kinetics. All these limit the application of the molecular networks in the identification of drug targets.

## 4. Identifying Drug Targets with Drug Effects

**Figure 3:**
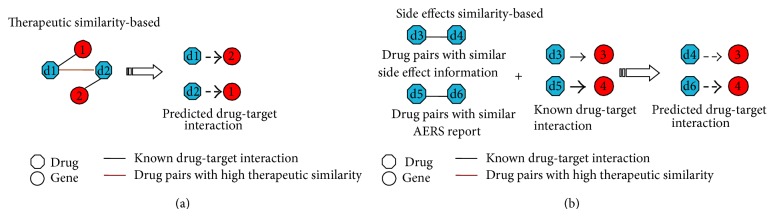
A schematic view of identifying drug-target interactions based on drug effect profiles. (a) Identify drug-target interaction based on therapy information by assuming that drugs with similar therapy may target same protein(s). (b) Predict drug targets based on side effects supposing that drugs with similar side effect have common target(s).

Except for the omics data from molecular space, a straightforward way to understand the drug MOAs is to explore the drug effects in the pharmacological space, which can in turn help predict the drug targets. Similar to the approaches based on gene expression profiles, the drug effect based approaches assume that the drugs with similar therapeutic effect may target the same protein(s) (see Figure 3). For example, Yamanishi et al. [10] found that the drug therapy information can better characterize drug targets compared against the commonly used chemical structure information. With the drug pharmacological information predicted with chemical structures, they significantly improved the prediction accuracy with a supervised bipartite graph model. Cheng et al. [40] integrated the chemical structure information with pharmacological information, to predict DTIs, and obtained promising results, where the drug therapeutic similarity they used was defined by Xu et al. [41] as shown below:

(3) $$\begin{array}{c} \text{TS}\left(d_{1},d_{2}\right)=\frac{\sum_{k=1}^{3}S_{k}\left(d_{1},d_{2}\right)}{n}, \end{array}$$

where *n* ranges from 1 to 5 and *S*_*k*_(*d*_1_, *d*_2_) is defined as

(4) $$\begin{array}{c} S_{k}\left(d_{1},d_{2}\right)=\frac{{\text{ATC}}_{k}\left(d_{1}\right)\cap {\text{ATC}}_{k}\left(d_{2}\right)}{{\text{ATC}}_{k}\left(d_{1}\right)\cup {\text{ATC}}_{k}\left(d_{2}\right)}, \end{array}$$

where ATC_*k*_(*d*) denotes all the ATC codes at the *k*th level of drug *d*. Note that a drug has five levels of ATC codes.

In contrast to the therapy information, little attention has been paid to the adverse effects caused by drugs when predicting the drug targets. It is known that the unexpected drug side effects may be caused because of the off-targets [42, 43] and these off-targets may help to predict therapeutic targets. Recently, Campillos et al. [44] proposed a novel approach to predict the drug targets based on the drug side effects, where they assumed that the drugs with similar side effects will share common target proteins. To calculate the drug similarity based on their side effect profiles, they first extracted drug associated adverse effects from FDA adverse event reporting system and formalized them with the Unified Medical Language System (UMLS) ontology [45]. The drug side effect information has been deposited in the resource of SIDER [46]. With the drug adverse reaction information, they discovered unexpected connections among drugs with different chemical structures and therapeutic indications. By integrating the chemical structures and side effects, they significantly improved the prediction accuracy and identified some novel predictions which otherwise will not be found with only chemical structures. In addition, some of their predictions were experimentally validated, implying the predictive power of the side effects. With novel targets identified for old drugs, new potential indications can be found for these known drugs. For instance, the authors found that the nervous system drugs pergolide, paroxetine, and fluoxetine share the same targets with the drug rabeprazole that is an approved drug for relieving duodenal ulcer symptoms and treating ulcerative gastroesophageal reflux disease, indicating that these drugs may be repositioned for treating new diseases. Due to the scarceness of drugs' side effect information, Takarabe et al. [47] proposed a new approach, to predict novel drug-target interactions by integrating pharmacological information from AERS (adverse event reporting system) and genomic information for proteins, and found some novel targets.

The pharmacological information associated with drugs provides an alternative way to predict drug targets and has been proved to be complementary with the commonly used molecular information, for example, genome sequence or transcriptome data. Unfortunately, the scarceness of drug package and adverse reaction information limits the application of above-mentioned approaches to those well studied drugs. In addition, the drug effects are determined by the molecular context of their target proteins and those drugs with similar effects may not share any target proteins in fact. For example, Brouwers et al. [48] found that the drug side effects are determined by the neighborhood of their targets in a PPIN, where the same neighborhood does not necessarily mean same target proteins.

## 5. Discussion and Conclusion

The identification of drug targets plays essential roles in understanding the drug MOAs and designing new drugs with expected therapy. In this review, we summarized the recent progress on computational methodologies that have been developed to identify drug-target interactions. We summarized some recent popular tools or algorithms for drug target prediction in Table 2. Furthermore, we categorized these approaches according to the high-throughput data on which they work. In particular, we focused on those approaches that explore transcriptome, molecular network, and drug effect data due to their public availability. The transcriptome data provides a snapshot of the whole-genome dynamics and can help understand the mechanisms of action of drugs. The transcriptome-driven computational approaches assume that the drugs with similar gene expression signature will target the same protein. However, it is not easy to define a robust gene signature due to the noise and batch effects inherited in the gene expression data. The molecular network provides the circuit context in which the drug targets work, which makes the network approaches promising. Unfortunately, the incompleteness of the network knowledge and the network dynamics induced by drugs limit the application of these methods. Compared with the molecular data, the drug therapy and side effect information are more difficult to get. Therefore, the integration of distinct types and complementary data will be a promising direction in the future.

**Figure 4:**
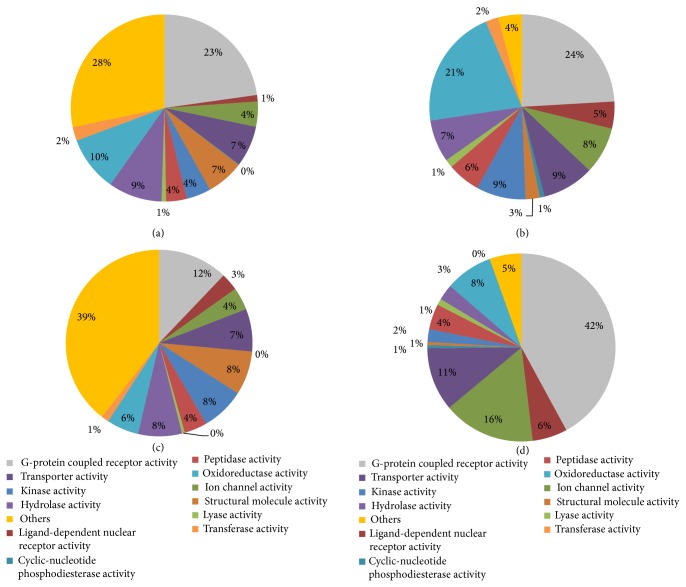
Functional distribution of human proteins (a), drug targets (b), neighbor proteins of drug targets (c) in the PPIN, and drug therapeutic targets (d).

Except for the above-mentioned data, the functions of the proteins targeted by drugs should also be taken into account.  Figure 4 shows the functional distribution of human proteins, drug targets, neighborhood proteins of drug targets in PPIN, and drug therapeutic targets. The drug target information was extracted from DrugBank [5] database, the neighborhood proteins are those direct neighbors of drug targets in the PPIN that was extracted from Entrez Gene Database [56], and the therapeutic targets were retrieved from [57]. All the proteins were grouped according to the molecular function annotations from Gene Ontology [58]. We can clearly see that the drug targets have different functions compared with the human genome background. On the other hand, the therapeutic targets have different functions from all proteins that can be targeted by drugs, implying that the off-targets may have specific functions. What is interesting is that unlike the drug targets, most of which belong to the GPCR family, the neighborhood proteins of drug targets belong to transferase. This information should be utilized to improve prediction accuracy when developing new methodologies in the future.

## Figures and Tables

**Table 1:** Popular drug target databases.

Drug target databases	Websites
DrugBank	http://www.drugbank.ca/
STITCH	http://stitch.embl.de/
ChEMBL	https://www.ebi.ac.uk/chembldb/
Superdrug	http://bioinformatics.charite.de/superdrug2_/
DGIdb	http://dgidb.genome.wustl.edu/
Binding DB	http://www.bindingdb.org/bind/index.jsp
CLiBE	http://xin.cz3.nus.edu.sg/group/clibe/clibe.asp
The TDR Targets database	http://tdrtargets.org/
Comparative Toxicogenomics Database (CTD)	http://ctdbase.org/
IUPHAR-DB	http://www.iuphar-db.org/index.jsp
PROMISCUOUS	http://bioinformatics.charite.de/promiscuous/
KEGG BRITE	http://www.genome.jp/kegg/brite.html
Potential Drug Target Database (PDTD)	http://www.dddc.ac.cn/pdtd/
Therapeutic Target Database (TTD)	http://bidd.nus.edu.sg/group/ttd/ttd.asp

**Table 2:** Popular software/algorithms for identifying drug target.

Reference	Data used
Iskar et al. [14]	Transcriptome profiles
Reinhold et al. [18]	Transcriptome profiles
Cheng et al. [21]	Transcriptome profiles
Carrella et al. [49]	Transcriptome profiles
Xu et al. [50]	Transcriptome profiles
Zhao and Li [26]	Molecular networks
Li et al. [35]	Molecular networks
Gönen [51]	Molecular networks
Wang et al. [52]	Molecular networks
Yizhak et al. [39]	Molecular networks
Yang et al. [53]	Molecular networks
Takarabe et al. [47]	Drug effects
Campillos et al. [44]	Drug effects
Mizutani et al. [54]	Drug effects
Iwata et al. [55]	Drug effects
